# Data Quality of Chinese Surveillance of COVID-19: Objective Analysis Based on WHO’s Situation Reports

**DOI:** 10.1177/1010539520927265

**Published:** 2020-05-14

**Authors:** Alvaro Javier Idrovo, Edgar Fabián Manrique-Hernández

**Affiliations:** 1 Universidad Industrial de Santander, Bucaramanga, Santander, Colombia

**Keywords:** coronavirus, pandemic, epidemiological surveillance, data quality, China

## Abstract

Was there quality in the Chinese epidemiological surveillance system during the COVID-19 pandemic? Using data of World Health Organization’s situation reports (until situation report 55), an objective analysis was realized to answer this important question. Fulfillment of Benford’s law (first digit law) is a rapid tool to suggest good data quality. Results suggest that China had an acceptable quality in its epidemiological surveillance system. Furthermore, more detailed and complete analyses could complement the evaluation of the Chinese surveillance system.

Good epidemiological surveillance systems are essential for epidemic management. One of its functions is to provide data with quality that serve to make decisions based on evidence.^1^ Unfortunately, it is not easy to know the quality of data during public health emergencies of international concern such as the COVID-19 pandemic. During the influenza A(H1N1) pandemic, Benford’s law was proposed as an objective and fast way to assess the performance of surveillance systems during epidemics.^2^ Its usefulness was also evidenced in the dengue epidemic in Paraguay (2009-2011)^3^ and the Zika epidemic in American countries.^4^

Benford’s law,^5^ also called “law of the first digits,” “Newcomb-Benford law,” or “law of anomalous numbers,” states that for a determined set of numbers, those whose first digit is 1 will appear more frequently (30.103%) than those beginning with other digits, following in order from 2 to 9 (17.609%, 12.494%, 9.691%, 7.918%, 6.695%, 5.799%, 5.115%, and 4.576%, respectively).^6^ A very good explanation of Benford’s law is the following:

“The good fit of the Newcomb-Benford law to empirical data can be explained by the fact that in many cases the frequency with which objects occur in “nature” is an inverse function of their size. Very small objects occur much more frequently than do small ones, which in turn occur more frequently than do large ones and so on.”^7^

The COVID-19 pandemic began in Wuhan (Hubei, China) in early December 2019.^8^ From there, it had a rapid spread through Asia, Europe, America, and Africa. At the end of March 2020, there were more than 850 000 cases and 40 000 deaths around the world.^9^ This brief report presents the results of an objective evaluation of data quality of the Chinese epidemiological surveillance system during the ongoing epidemic.

To obtain evidence on the level of performance of the Chinese epidemiological surveillance system, we used data included in situations reports 1 to 55 of the World Health Organization (WHO) website (January 21 to March 15, 2020).^10^ In these situational reports are found the number of confirmed cases, suspected cases, and deaths in the past 24 hours, and cumulated confirmed cases and deaths, in each Chinese province, region, and city. Given that by March 16, the numbers of cases and deaths outside China had overtaken the total number of cases in China, the WHO decided not to report separately the situation in the Chinese territories.

Cumulative cases reported by Chinese provinces, regions, and cities were evaluated according to how closely they followed the distribution of Benford’s law using log-likelihood ratio test. Results of these analyses are presented in Table 1. As can be seen, in the first situation reports, the information did not maintain a standard nor did the data show stable quality. Since the situation report 13, the data improved its quality in a more stable way. In Figure 1 is the Benford distribution of the last situation report analyzed, and a detailed analysis of each digit with the χ^2^ test (Table 2).

**Table 1.** Fulfillment of Benford’s Law of Chinese Epidemiological Surveillance System of COVID-19 (Cumulative Confirmed Cases).

Situation Reports	n	Log-Likelihood Ratio, *P*			χ^2^, *P*		
		<.05	.05 to .10^a^	>.10^a^	<.05	.05 to .10^a^	>.10^a^
1	4	Excluded; few data					
2	14	√					√
3 and 4	17			√			√
5 to 12	1	Only all country data available					
13 to 33	34			√			√
34 and 35	34		√			√	
36 and 37	34			√			√
38 to 42	34		√			√	
43 to 55	34			√			√

a Fulfillment of Benford’s law.

**Figure 1.**
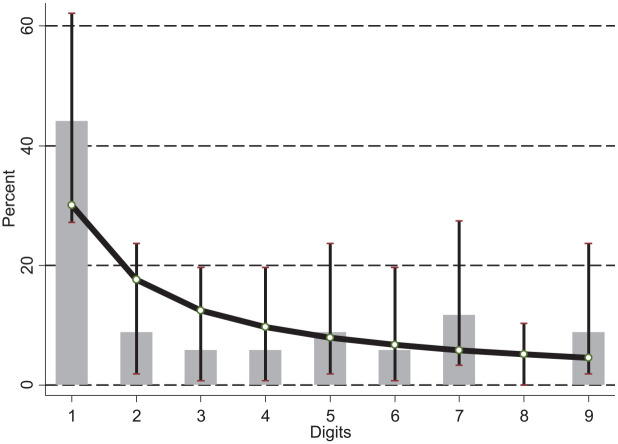
First digit frequencies for the Benford distribution of COVID-19 epidemic situation report 55 in China (March 15, 2020).

**Table 2.** Details of Fulfillment of Benford’s Law of Chinese Epidemiological Surveillance System of COVID-19 (Cumulative Confirmed Cases) in Situation Report 55.

First Digit	Count	Percentage		*P*
		Observed	Expected	
1	15	44.118	30.103	.0912
2	3	8.824	17.609	.2580
3	2	5.882	12.494	.4309
4	2	5.882	9.691	.7689
5	3	8.824	7.918	.7489
6	2	5.882	6.695	1
7	4	11.765	5.799	.1322
8	0	0	5.115	.4190
9	3	8.824	4.576	.2025

The results suggest that the Chinese epidemiological surveillance system has had good data quality during the current health emergency. This must be understood in a pandemic context, and being the country where it originated. In these circumstances, it is highly probable that the reported data are underestimated in great magnitudes, as presented in recent publications.^11^ It is frequent in these contexts that only most severe cases are reported. With well-designed population studies, the numerator and denominator of the proportions of the actual occurrence of the infection will be better known. Previous experiences such as Lassa fever in Africa suggests that infections with initial high fatality diminish the severity with widespread epidemiological studies.^12^

However, this evaluation did not incorporate all the elements of the health system involved in the management of the current COVID-19 pandemic. A comprehensive evaluation of the Chinese surveillance system should add to the data quality, characteristics of simplicity, flexibility, acceptability, sensitivity, positive predictive value, representativeness, timeliness, and stability.^1^ However, this rapid evaluation serves to provide feedback to officials of the Chinese surveillance system. This type of ongoing evaluation is possible in other countries, and it will allow officials in charge to make relevant decisions to improve epidemiological surveillance systems and the response of health care services.
